# Memoirs of the Royal Academy of Sciences at Paris, for the Year 1780

**Published:** 1785

**Authors:** 


II. *Memoirs of the Royal Academy of Sciences at Paris, for the Year* 1780. (Continued from page 184.)

IX. *REMARKS on the Combination of fixed Alkali with fixed Air. By* M. Bertholet.—Fixed air, we are here informed, has the property of chryſtallizing fixed alkali: hence, if concrete volatile alkali is mixed with fixed alkali, the latter chryſtallizes, while the former mer is rendered more cauftic by being deprived of its fixed air.

If fixed alkali is diffolved in nitre faturated with fixed air, and marine falt with an earthy bafis is added to the folution, no precipitation will, in general, be perceptible. This has led to a fuppofition that no decompofition takes place; but M. Beitholet has obferved that in this cafe there is a double decompofition, and that a combination of the fixed air and earth remains in the folution.

X. *Obfervations on the Effect produced on fine Gold by the nitrous Acid.* *By* M. Tillet.

XI. *Remarks on the Structure and Alterations of the Glands of the Lungs; and on the Nature of certain Symptoms of Phthifis Pulmonalis.* *By* M. Portal.— Thefe remarks on the glands of the lungs are founded on a fuppofition, which we cannot admit, that the bronchial glands, as they are called, are different from the lymphatic glands of thofe organs.

The change which fometimes takes place in the voice, in cafes of phthifis, is attributed by our author to the irritation of the recurrent nerves occafioned by tubercles or abfceffes in the lungs. Thus in the cafe of a lady, we are told, who at firft complained of a pain in her throat, throat, and afterwards of a loſs of voice, and who died in an emaciated ſtate, on diſſection, no morbid appearance was diſcovered in the larynx, but an abſceſs and tubercles were found at the top of the left lobe of the lungs, and at the part where the recurrent nerves ſend off branches to thoſe organs. In another lady, whoſe caſe is related, and who laboured under great difficulty of deglutition, which terminated fatally, the voice was much affected, being at firſt more acute, afterwards hoarſe, and at length totally extinct. After her death M. Portal found the organs of voice and deglutition in a natural ſtate; but he obſerved marks of conſiderable inflammation at that part of the pericardium which receives numerous branches of the recurrent nerves.

XII. *On certain Fluids which may be procured in an aeriform State, at a Degree of Heat a little above the mean Temperature of the Earth. By* M. Lavoiſier.

XIII. *A ſecond Eſſay on the different Combinations of the phoſphoric Acid. By* the ſame.— This is a continuation of an inquiry begun in a former volume*. In the preſent paper the au-

* See our ſecond vol. page 106.

thor gives the refults of his experiments on the combination of this acid with fpirit of wine and different metals. He has not yet been able, it feems, to procure phofphoric æther. The phofphoric acid, we are told, diffolves iron, but has no effect on gold; and its action on copper is faid not to exceed that of the nitrous acid on gold.

XIV. *An Account of a particular Procefs for converting Phofphorus into phofphoric Acid without Combuftion. By* the fame. — This procefs confifts in throwing phofphorus, by little and little, into a retort containing nitrous acid, and diftilling it by a gradual increafe of heat. In the ebullition fmoking fpirit of nitre comes over, and the acid of phofphorus remains in the retort.

XV. *An Effay on Heat*, by Meffieurs* Lavoifier *and* de la Place.

XVI. *Report concerning the State of the Prifons. By Meffieurs* du Hamel, de Montigny, le Roy, Tenon, Tillet, *and* Lavoifier.

XVII. *Remarks on the Infirmaries of the three principal Prifons within the Jurifdiction of the Chatelet at Paris. By* M. Tenon.

* See Vol. IV. page 235.

XVIII. *On*

XVIII. *On the Caufticity of metallic Salts. By* M. Bertholet.

XIX. *Anatomical Remarks on three Species of Monkey, called, by M. de Buffon, the* Mandrill, *the* Callitriche, *and the* Macaque ; *to which are added fome Reflections on different Points of comparative Anatomy. By* M. Vicq D'Azyr. — The diffection of the fecond of thefe fpecies afforded the author an opportunity of obferving a fingular ftructure of the heart, the thicknefs of the parietes of the left ventricle being nearly five times greater than that of the right.

XX. *Refearches concerning the Structure and Pofition of the Tefticles in the abdominal Cavity of the Fœtus; their Paffage out of the Belly; and the Obliteration of the Tunica Vaginalis: intended as a Supplement to the Obfervations publifhed in* 1762, *on the fame Subject, by Mr. John Hunter. By* M. Vicq D'Azyr.—It was Mery, according to our author, who, in the Memoirs of the Academy for 1701, firft defcribed a hernia in which the tefticle and inteftine were in contact. He gives to Haller the credit of having firft known the true formation of this fpecies of hernia, as well as the fituation of the tefticles in, and their defcent from the abdomen in the fœtus. M. Vicq D'Azyr finds that in the fifth or or fixth month after conception the body of the epididymis is detached from the tefticle to which it adheres only by its two extremities. He obferves, that the opening which eftablifhes a communication between the tunica vaginalis and abdomen, clofes in two places, and by two different proceffes: 1ft, a thin membrane continued from the peritonæum clofes the ring, and forms on the fide of the belly a fmooth polifhed furface; 2dly, an obliteration takes place below this, which leaves between the tunica vaginalis and the ring a pretty confiderable fpace occupied by a fpecies of chord, which is placed more fuperficially than that of the fpermatic veffels. This appearance, which is fometimes to be met with in the adult ftate, is no other, he obferves, than the portion of tunica vaginalis obliterated after the complete defcent of the teftis. Thefe defcriptions are illuftrated by engravings.

XXI. *On the fpontaneous Inflammation of Phofphorus: with fome Remarks on the Nature of its Acid. By Meffieurs* de Laffone *and* Cornette. — Thefe gentlemen found that, by wafhing phofphorus in very cold water, it inflames fpontaneoufly. They attribute this phænomenon non to the heat generated by mixing water with the phoſphoric acid.

XXII. *On the Action of vitriolic Acids on Oils. By* M. Cornette.—Theſe experiments prove that as many ſpecies of ſoap may be formed with vitriolic acid as there are different kinds of oil, whether eſſential or expreſſed.

XXIII. *On the Action of marine Acid on Oils. By* the ſame.—The marine acid, we here learn, though concentrated, acts too feebly on oils to form ſoap. It keeps, however, a ſmall quantity of the oil in ſolution, and thus, in a certain degree, renders it miſcible with water; and expreſſed oil, which has been ſubmitted more than once to the action of the acid, becomes ſoluble in ſpirit of wine, and acquires a reſinous property. The ſame acid, mixed with eſſential oil of lavender, and diſtilled, yields the ſame odour as the acid ſpirit of amber. M. Cornette adds, that he has procured marine ſalt from amber, a fact which he thinks ſeems to confirm an opinion of the late M. Bourdelin, that the acid of amber is a marine acid modified by ſome unknown principle.

XXIV. *On the Changes which eſſential and expreſſed Oils undergo from the Action of the nitrous Acid. By* the ſame.—Nitrous acid inflames eſſential effential oils; but M. Cornette finds that it requires to be mixed with vitriolic acid to produce the fame effect on expreffed oils. Thefe laft, he obferves, are only feebly attacked by the nitrous acid, but it has a fufficient effect to render them foluble in fpirit of wine.

XXV. *Experiments on fedative, nitrous, marine, and acetous Salts; made with a View to afcertain the Difference between thofe Salts, which have hitherto been fuppofed to be of the fame Nature. By* M. Cadet.

XXVI. *Remarks on the thoracic Duct of the human Body. By* M. Sabatier.—We have here a very accurate account of the ftructure of this duct, and of the varieties that have occurred to the author in the diffection of a great number of different fubjects.

XXVII. *On fome eafy Methods of renewing the Air in Places where it either does not circulate, or circulates with Difficulty; and on the Ufes to which they may be applied. By* M. le Roy.

XXVIII. *Botanico—Meteorological Obfervations made in the Year* 1779. *By* M. du Hamel.

XXIX. *On the different Species of Dog Fifh. By* M. Brouffonet.

III. *A prac-*

